# An assessment of data and code sharing in research funded by the Nathan Shock Centers, 2017–2022: a meta-research study

**DOI:** 10.1093/geroni/igag090

**Published:** 2026-08-13

**Authors:** Colby J Vorland, Simon Chung, Aidan S Kramer, John R Hultquist, David B Allison, Andrew W Brown

**Affiliations:** Department of Epidemiology and Biostatistics, School of Public Health-Bloomington, Indiana University, Bloomington, Indiana, United States; Department of Biostatistics, University of Arkansas for Medical Sciences, Little Rock, Arkansas, United States; Arkansas Children’s Research Institute, Little Rock, Arkansas, United States; School of Public Health-Bloomington, Indiana University, Bloomington, Indiana, United States; School of Public Health-Bloomington, Indiana University, Bloomington, Indiana, United States; Children’s Nutrition Research Center, Baylor College of Medicine, Houston, Texas, United States; Department of Biostatistics, University of Arkansas for Medical Sciences, Little Rock, Arkansas, United States; Arkansas Children’s Research Institute, Little Rock, Arkansas, United States

**Keywords:** Reproducibility, Replicability, Nathan Shock Centers

## Abstract

**Background and Objectives:**

Data and code sharing facilitate reproducibility, trust, and efficient reuse of research resources. Growing recognition of their importance among researchers, journals, and funders led the Nathan Shock Centers (NSC) Coordinating Center to propose documenting and supporting data and code sharing in aging research. As a baseline, we characterized data and code sharing practices in NSC-funded research.

**Research Design and Methods:**

We surveyed articles citing NSC grants published 2017–2022 indexed in PubMed. In full-text screening (*n *= 507), we excluded articles that did not generate or analyze data. For included articles, we classified data/code availability statements as available via repository, supplemental file, inclusion in paper, available on request, explicitly not available, no statement included, or other. We checked articles indicating open data/code to determine whether materials could be located per the provided statement.

**Results:**

Of 400 articles included, 50% and 92% had no data or code availability statements, respectively. Data statements indicated availability via repository (30%), supplemental files (26%), inclusion in paper (10%), and on request (14%). For code, 6% indicated availability in repositories, and ≤1% each for other categories. Of those indicating open data or code, materials were located for 63% and 81%, respectively.

**Discussion and Implications:**

Availability statements were absent for about half of articles for data and most for code; when open data or code was claimed, materials were located in most, but not all. Future work will evaluate how these practices improve over time as the NSC Coordinating Center’s support and journal and funder guidelines evolve.

Innovation and Translational Significance: Data and code sharing support the verification and reuse of research findings, yet the extent of these practices in aging research has been unknown. In a systematic assessment of 400 articles funded by the Nathan Shock Centers (2017**–**2022), half of articles included no data statement, and most (92%) included no code statement; where open data or code was indicated, the underlying materials could be located in most, but not all. Characterizing these practices provides a baseline against which data and code sharing can be improved, supporting greater transparency, verification of findings, and reuse of existing data in aging research.

## Background and objectives

Data and code sharing are vital to the trustworthiness of scientific research by enabling transparency of methods and facilitating computational reproducibility and verifiability of results. The recognition of the importance of data and code sharing is becoming more salient, evidenced by new policies. For instance, the National Institutes of Health (NIH) updated its Data Management and Sharing Policy in 2023 to require that researchers prospectively plan for data sharing (National Institutes of Health). Similarly, the White House Office of Science and Technology Policy guidance mandated that all federally funded research agencies update policies by the end of 2025, with plans to make data supported by public funds freely available (Office of Science and Technology Policy, 2022).

Data and code sharing practices have been studied in many domains across medical research. In a systematic review and meta-analysis of 105 meta-research studies, the mean and actual public data availability between 2016 and 2021 was 8% and 2%, respectively (Hamilton et al., 2023). The prevalence of declared and actual code availability was estimated at <0.5%. Actual data and code availability did not increase over time, though declared data availability increased. An automated assessment of 2.75 million articles published on PubMed Central estimated that, on average, data sharing was mentioned in 14.5% of articles and code sharing in 2.5% of articles (Serghiou et al., 2021). These results suggest that data and code sharing practices remain low across research domains; however, it is not known whether sharing practices among researchers in aging biology are consistent with these trends.

The Nathan Shock Centers (NSC) of Excellence in the Basic Biology of Aging provide funding opportunities for researchers investigating the basic biology of aging, and at the time of this study, were hosted within eight institutions. The supporting grants are required to be listed in any resulting published articles, and as such, provide a convenient sample of aging articles to evaluate for data and code sharing practices. The objective of this study was to assess the current state of data and code sharing practices in published research funded by NSC. By conducting a systematic assessment of data and code sharing, we provide a snapshot of data and code sharing practices in a cohort of researchers who study aging. This assessment was conceived as an introspective baseline by researchers affiliated with the NSC network and its Coordinating Center to establish a starting point for improvement rather than to evaluate any individual investigator, article, or center.

## Research design and methods

### Corpus

To obtain a subset of aging-specific studies, we obtained all articles listed on PubMed over a 5-year period with publication dates from April 1, 2017 (the project start date of the NIH Nathan Shock Center Coordinating Center grant; U24AG056053) to April 1, 2022, that were linked to a grant number affiliated with a Nathan Shock Center (search string is provided in Supplementary Material). The search was run on April 7, 2022. Search results were imported into EndNote to locate institutionally linked full texts, and the rest were found manually. The renewal of the NSC Coordinating Center includes dedicated support for improving data sharing practices, so the current corpus was chosen as a baseline for future assessments of whether data sharing practices change throughout the Coordinating Center renewal period.

### Screening

Full texts were imported into Sysrev (Bozada et al., 2021) for screening in duplicate at all steps (C.J.V., A.S.K., and J.R.H.).

We screened all full texts and followed the following convention when screening:

Inclusion criteria (all required for inclusion): (a) indexed in PubMed with a publication date between April 1, 2017, and April 1, 2022; (b) linked to at least one NSC-affiliated grant number; and (c) an original research article that generated or analyzed data such that a data- and/or code-availability assessment was applicable.

Exclusion criteria: article types that did not generate or analyze data (e.g., reviews, editorials, opinions).

With all included articles, as a first step, we classified data and code availability statements according to what the authors wrote in their papers (Table 1). Articles could contain multiple data and/or code availability classifications.

**Table 1:** Categories used to classify data and code availability.

Data availability	Code availability
Data are available in a repository Data are available in a supplementary/supporting file(s) (e.g., a .csv file) Data are included in the paper Data are available upon request There is an explicit statement that data will not be made available There is no statement about data in the paper Other (add note in textbox for discussion)	Code is available in a repository Code is available in a supplementary/supporting file(s) (e.g., a Word or notepad file) Code is included in the paper Code is available upon request There is an explicit statement that code will not be made available There is no statement about code in the paper Other (add note in textbox for discussion)

In the context of this study, we defined data as the individual-level measurements (“raw data”) necessary to independently computationally reproduce findings of the article, as distinct from summarized or aggregated data. Similarly, we defined code as the statistical source code required to process the raw data and generate results.

For articles that indicated that data or code were openly available (i.e., in a repository, supplementary/supporting file(s), or included in the paper), we then sought to confirm whether any raw data or code were present from that source to confirm whether we could find them. We classified raw data or code as confirmed if we could locate individual-level subject data that would be needed to reproduce at least some outcomes, and statistical code that would be required to reproduce the analysis of at least some of the results. If the data availability statement indicated that data were included in the paper, we checked both the main publication and sought and screened any supplementary/supporting file(s). If the data or code statement indicated availability in a repository, we followed links or searched repositories using article metadata to attempt to locate them; if the repository required a free account, we created an account, but any paid access was classified as not confirmed.

For the purposes of this study, we defined repository broadly to include any publicly available website where data or code was hosted, including institutional or personal websites. We acknowledge that others may require a characteristic of a repository to have a commitment to long-term preservation and access, assignment of persistent unique identifiers, and metadata.

Herein, we follow the recommended adaptations for PRISMA reporting guidelines for meta-epidemiological studies (Murad & Wang, 2017).

## Results

### Characteristics of included publications

**Figure 1:**
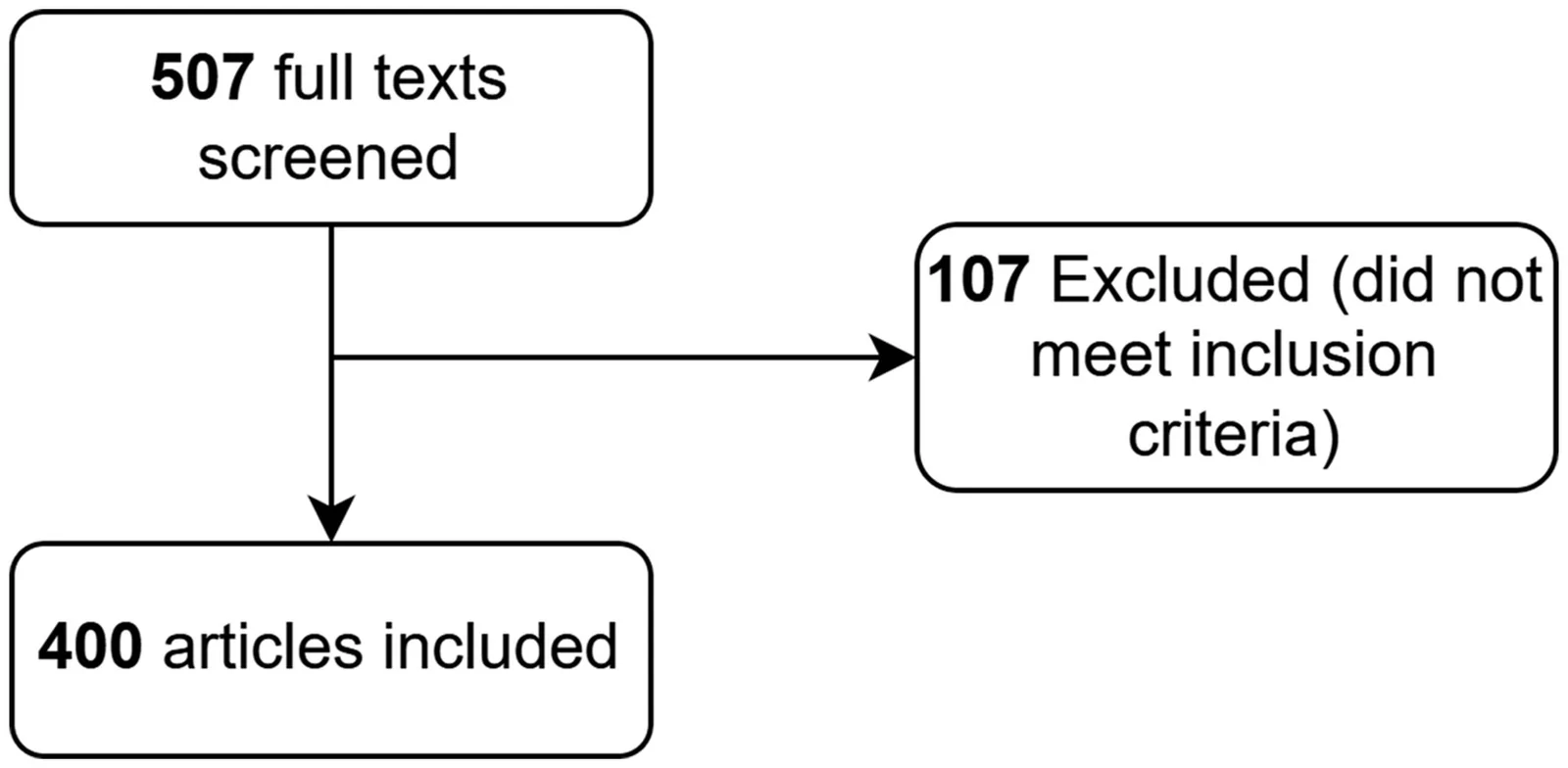
Flow diagram of article screening.

The search yielded 507 results and excluded 107 (Figure 1). Of the 400 remaining articles, 59 (15%) were published in 2017 (from April), 75 (19%) in 2018, 79 (20%) in 2019, 83 (21%) in 2020, 87 (22%) in 2021, and 17 (4%) in 2022 (until April). Articles were published in 155 different journals, most often published in the journals *Geroscience, Aging Cell, The Journals of Gerontology: Series A*, and *Redox Biology* (Supplementary Material). The median number of authors per article was 9 (IQR = 6). The median number of authors per article was 9 (IQR 7–13). Reflecting this broad authorship, the 400 articles were written by 2,768 unique authors (using last name and first initial to disambiguate names), including 328 distinct first authors and 216 distinct last authors.

Research in the corpus spanned research models including cells (e.g., macrophages, dendritic cells), dogs, drosophila, elephants, rodents (e.g., strains of mice and rats), and human research from children to older adults and from observational to trials. Methodological and modeling studies were also obtained.

### Data availability

**Figure 2:**
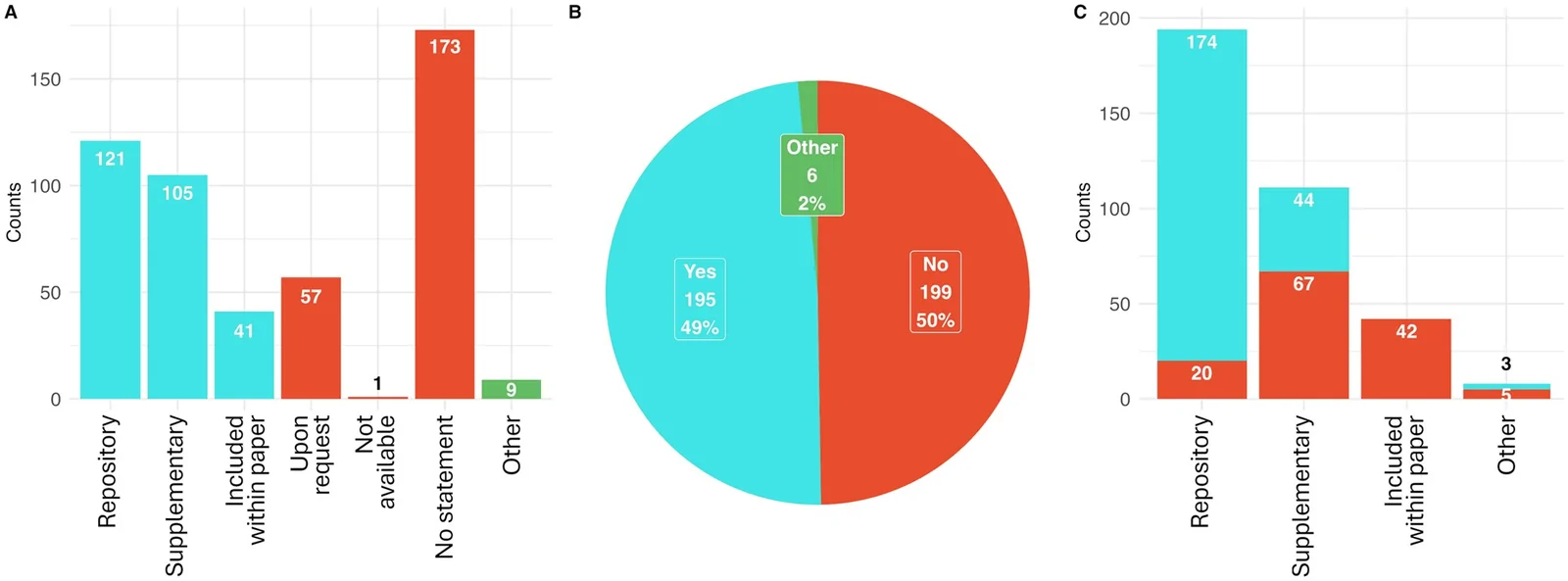
Data availability and accessibility in sampled articles. (A) Frequency of different data availability statements (blue [bars 1, 2, and 3] = stated publicly available, red [bars 4, 5, and 6] = stated not publicly available or no statement, green [bar 7] = other). Articles can be rated with more than one category, but only once per category (e.g., multiple repositories only count as repository once within article), so the sum of ratings is greater than the 400 included articles. (B) Proportion of articles that claimed data was publicly available (red = no, blue = yes, green = other). Articles are counted only once. (C) Frequency of accessible individual data when data availability statements claimed publicly available data, by location of claim (red [bottom] = not accessible, blue [top] = accessible). Bars represent counts of all sources in the 400 articles. Articles may have more than one source within a category (e.g., two repositories), and availability is assessed separately for each source.

Fifty percent of articles (*n *= 199) contained no statement about data availability (Figure 2A), followed by stating that data were available in a repository, in supplementary/supporting file(s), available upon request, included in the paper, and “other,” respectively. One article contained an explicit statement that data would not be made available.

Of the 400 articles, 195 (49%) stated that data were publicly available (Figure 2B). Of these 195 articles, data were located 63% of the time. Data were located most of the time (90%) when they were stated to be in a repository (Figure 2C). When data were stated to be in a supplementary/supporting file(s), data were located 40% of the time. When the statement indicated that data were included in the paper, individual-level data were not found. When the statement was classified as other, data were located 38% of the time.

### Code availability

**Figure 3:**
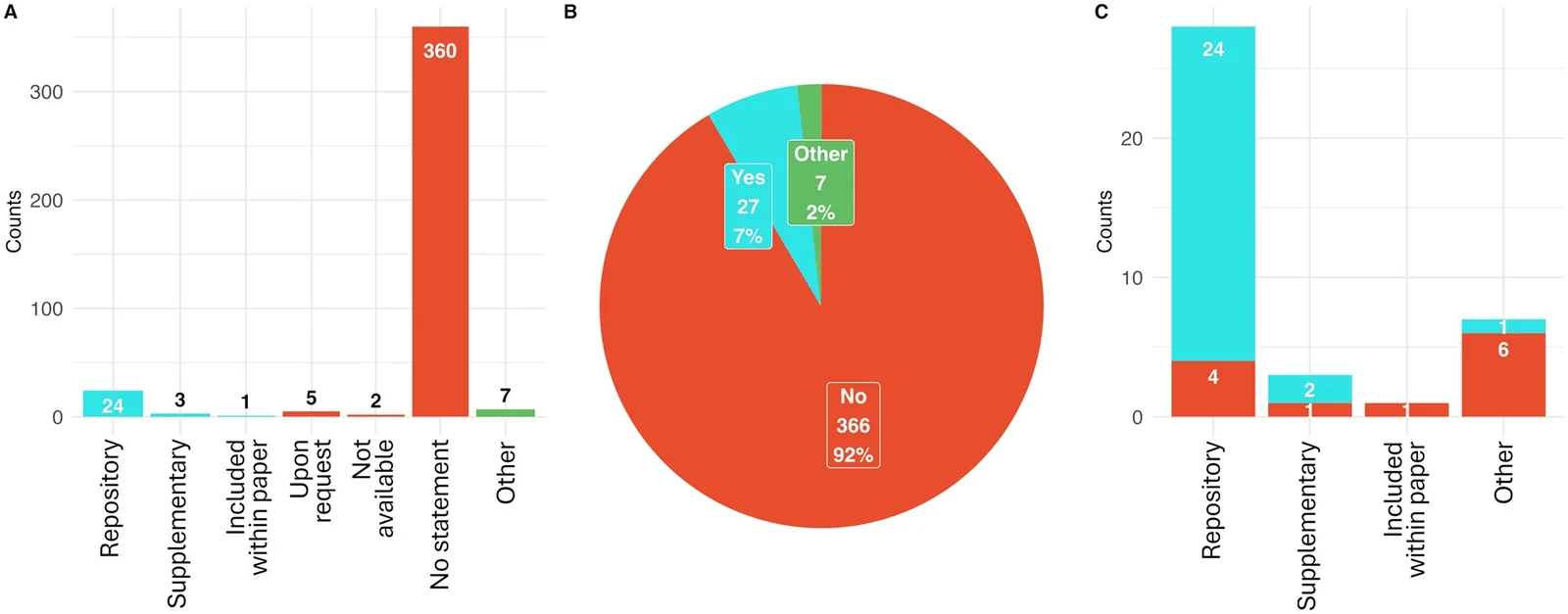
Code availability and accessibility in sampled articles. (A) Frequency of statistical code availability statements (blue [bars 1, 2, and 3] = stated publicly available, red [bars 4, 5, and 6] = stated not publicly available or no statement, green [bar 7] = other). Articles can be rated with more than one category, but only once per category (e.g., multiple repositories only count as repository once within article), so the sum of ratings is greater than 400. (B) Proportion of articles that claimed code was publicly available (red = no, blue = yes, green = other). Each article is counted only once. (C) Frequency of accessible code when code availability statements claimed publicly available code, by location of claim (red [bottom] = not accessible, blue [top] = accessible). Bars represent counts of all sources in the 400 articles. Articles may have more than one source within a category (e.g., two repositories), and availability is assessed separately for each source.

Ninety-two percent of articles contained no statement about code availability (Figure 3A). Some articles stated that code was available in a repository, and a few stated that code is available on request, in a supplementary/supporting file(s), or is included in the paper. Two articles explicitly stated that code will not be made available.

Of the 400 articles, 27 (7%) stated that code was publicly available (Figure 3B), and overall, we could locate code 81% of the time. Code could be located most of the time (86%) when it was stated to be in a repository, 67% of the time when stated to be in a supplementary/supporting file(s), 14% of the time when classified as “other,” and was not located for the one article stating that code was included in the paper (Figure 3C).

### Case studies

There are several notable case studies within the articles we sampled that are worth highlighting for the community. One article claimed to analyze data using code availability on the website “wikiflic.com,” which reflected a “Fly Liquid-food Interaction Counter (FLIC) system.” At the time of screening, the domain had expired and redirected to a website advertising lewd images. At the time of submission of this manuscript, nine articles referenced “wikiflic.com,” per Google Scholar. One article directed users to a lab website for additional files, where we could not identify raw data or code. It is possible that files existed at one point, but the website changed. Both examples highlight the importance of stable data and code repositories (Attwood et al., 2015; Mannocci et al., 2022; Strecker et al., 2023).

## Discussion and implications

In this systematic survey of articles funded by the NSC of Excellence in the Basic Biology of Aging between 2017 and 2022, half of the articles lacked a data availability statement, most lacked a code availability statement, and open data or code could not always be located.

A substantial portion of articles included no statement on data availability (50%) or code availability (92%). Even when availability was indicated, access to the underlying materials was not guaranteed, as we were able to confirm the availability of open data and code in 63% and 81% of those cases, respectively. These findings are broadly consistent with, though in some respects more optimistic than, recent meta-research across other biomedical fields, which has also documented low rates of actual data and code sharing despite increasing calls for transparency. The push for open science principles is driven by the recognition that access to underlying data and code allows for the verification of findings and potentially the acceleration of discovery through the reuse of existing data.

Hamilton and colleagues synthesized 105 meta-research studies of over 2.1 million articles that investigated data and code sharing in medical and health sciences (Hamilton et al., 2023), providing a benchmark for sharing. They estimated that 8% of articles included a statement stating that data were publicly available, and only 2% actually shared data. For analytical code, both stated and actual public availability rates were estimated to be less than 0.5%. Against these averages, NSC sharing practices are a step ahead. There may be myriad reasons for this, such as different methods of classifying data and code statements, the focus on a sample of papers within geroscience research, or that the culture of sharing within this subdiscipline is higher because of the dedicated resources for doing so afforded by the NSCs. Such reasons are an area of future research. The assessment presented here provides a critical baseline for the NSC Coordinating Center, which has been funded to document and support the improvement of these practices over time during its renewal period.

A key factor influencing sharing practices is the journal in which the research is published. Journals have widely varying policies and enforcement mechanisms for data and code availability, which are known to be significant drivers of author behavior. The present journal, for instance, indicates that authors should state whether data, analytic methods or materials are available, and, if so, where, but only “encourages, but does not require” sharing (The Gerontological Society of America, 2026). A Nature Research data availability statement guide from 2016 (Nature Research, 2016), still actively linked in some journals and resources, gives examples of data availability statements, some of which are useful and some not. We highlight three in the context of our results here, but note that many journals or publishers use similar statements (e.g., Wiley’s Data Sharing Policies, 2026).

One helpful statement template is as follows: “The datasets generated during and/or analysed during the current study are available in the [NAME] repository, [PERSISTENT WEB LINK TO DATASETS].” Such a templated statement leads investigators to point to where their data reside, and we had the most success accessing data and code in named repositories.

One modestly helpful statement template states, “All data generated or analysed during this study are included in this published article (and its supplementary information files).” If the data are genuinely available with the manuscript, then this statement is helpful. However, our results indicate that over half of the papers in our corpus that made such a statement did not contain all of the data within a supplement, and none within the paper. It is unclear whether authors are genuinely confused that summary statistics and results are not data, or whether they selected a data availability statement just to advance through review. Regardless, it is incumbent on editors and reviewers to confirm that data are available as stated by the authors.

Next, we share an unhelpful statement template: “The datasets generated during and/or analysed during the current study are available from the corresponding author on reasonable request.” Gabelica et al. (2022) demonstrated the abject failure of “data available upon request” statements: they requested data from authors of nearly 1,800 publications in which they declared data available on reasonable request, and received data in only 6.8% of cases. Expansions on the “upon request” statements that include steps required to access data are helpful if there are genuine reasons for not making data available to inform the reader of whether there will need to be data use agreements, third-party permissions, or human subjects protections. Authors could be required to demonstrate that such shareable materials are able to be produced prior to final acceptance or publication.

### Strengths, limitations, and further context

This study has several strengths. We analyzed the entire population of NSC-funded articles indexed in PubMed during the 5-year period. Each paper was manually assessed, including identifying and confirming whether data or code were available where studies indicated that data or code should be.

A limitation, however, is that our search was confined to a specific time window and a single database, although PubMed is a standard for this field. NIH-funded research is required to be included in PubMed Central by the Public Access Policy, effective since 2008, though inclusion in our sample depends on compliance with that policy. In addition, future research could verify whether the identified data and code are complete and sufficient to reproduce all reported outcomes, though to do so is a substantial undertaking (Jamshidi-Naeini et al., 2026). Herein, we confirmed only that some files appearing to be raw data or code were accessible; we did not assess completeness. Thus, our findings likely overestimate the true rate of data and code availability needed for reproducibility of all findings. Lastly, we did not preregister our study, given the initial introspective focus on NSC improvement, and recommend that future research on this topic do so.

In our study, we did not stratify our analysis by journal, so it remains unclear to what extent our findings reflect the norms of the authors and their institutions versus the requirements of the journals they selected. Future work could explore the influence of specific journal mandates on sharing rates within the aging research community.

Furthermore, heterogeneity may exist within the NSC themselves. Each center has a distinct scientific focus, includes specialized research cores, and may emphasize different research models (e.g., animals or humans); furthermore, subdisciplines may have different sharing norms. We deliberately chose not to summarize our findings by individual NSC. Our goal was to provide a network-wide assessment and avoid singling out specific centers; given the collaborative nature of research and the diverse scientific domains represented, trying to disaggregate by center could be misleading.

Finally, this assessment addressed only data and code sharing. These are necessary but not sufficient for reproducible research: complementary practices such as protocol sharing, Research Resource Identifiers, and reporting guidelines were outside our scope and represent additional targets for future evaluation and support.

### Implications

The challenges identified herein are not unrecognized by the NSC network. For instance, the University of Alabama at Birmingham NSC has contained the “Comparative Data Analytics Core,” of which several of the authors are or have been members, which supports investigators in facilitating data and code sharing. For already-published work, the verifiability of the record can be strengthened through several low-burden steps: depositing underlying data and code in a persistent, identifier-issuing repository (e.g., Zenodo, Open Science Framework, Dryad, or domain-specific repositories); and adding or correcting a data or code availability statement, for example, through a post-publication correction or linking on PubPeer.com. The NSC Coordinating Center is well positioned to support investigators in taking these steps. Such support is vital for improving standards. As the 2023 NIH Data Management and Sharing Policy (National Institutes of Health, 2023) and other federal guidance take full effect, we expect to see a positive shift in the practices documented here. This policy shift away from gated sharing models is critical, as demonstrated by the failure of the “data available on request” model (Gabelica et al., 2022). Our study provides the necessary benchmark against which future progress can be measured, offering a roadmap for targeted improvements in research transparency and reproducibility in the basic biology of aging. We emphasize that this is a benchmark, not a list of non-compliant articles. Our survey was done prior to any of the recent sharing policy changes, and therefore, none of the articles included in our sample were subject to these changes. Ultimately, open science practices are fundamental to the credibility and reproducibility of aging research (Isaacowitz & Lind, 2019).

## Supplementary Material

igag090_Supplementary_Data

## Data Availability

All data and code are available at https://doi.org/10.17605/OSF.IO/YQ2MA.
