# Supplementary material for: An assessment of data and code sharing in research funded by the Nathan Shock Centers, 2017–2022: a meta-research study
Source: Innov Aging. 2026 Aug 13;10(10):igag090. doi: 10.1093/geroni/igag090 (PMC13592470; doi:10.1093/geroni/igag090)
Supplement: Supplementary file 1 [file igag090_supplementary_data.pdf]

Innovation in Aging Supplementary Material: **Vorland et al. An assessment of data and code sharing in research funded by the Nathan Shock Centers, 2017-2022: A meta-research study.**

Search String

("2017/04/01"[Date - Publication] : "2022/04/01"[Date - Publication]) AND ((P30AG013280[Grant Number]) OR (P30 AG013280[Grant Number]) OR (AG013280[Grant Number]) OR (P30AG038072[Grant Number]) OR (P30 AG038072[Grant Number]) OR (AG038072[Grant Number]) OR (P30AG068345[Grant Number]) OR (P30 AG068345[Grant Number]) OR (AG068345[Grant Number]) OR (P30AG050911[Grant Number]) OR (P30 AG050911[Grant Number]) OR (AG050911[Grant Number]) OR (P30AG068635[Grant Number]) OR (P30 AG068635[Grant Number]) OR (AG068635[Grant Number]) OR (P30AG038070[Grant Number]) OR (P30 AG038070[Grant Number]) OR (AG038070[Grant Number]) OR (P30AG013319[Grant Number]) OR (P30 AG013319[Grant Number]) OR (AG013319[Grant Number]) OR (P30AG050886[Grant Number]) OR (P30 AG050886[Grant Number]) OR (AG050886[Grant Number]) OR (P30AG013283[Grant Number]) OR (P30 AG013283[Grant Number]) OR (AG013283[Grant Number]) OR (P30AG018254[Grant Number]) OR (P30 AG018254[Grant Number]) OR (AG018254[Grant Number]) OR (P30AG013314[Grant Number]) OR (P30 AG013314[Grant Number]) OR (AG013314[Grant Number]) OR (U24AG056053[Grant Number]) OR (U24 AG056053[Grant Number]) OR (AG056053[Grant Number]))

Frequency of journals in which articles were published

geroscience: 51
aging cell: 39
j gerontol a biol sci med sci: 19
redox biol: 18
elife: 14
nat commun: 11
aging (albany ny): 11
sci rep: 9
cell rep: 6
plos one: 6
cells: 5
neurobiol aging: 5
nat med: 4
j biol chem: 4
autophagy: 4
cell metab: 4
obesity (silver spring): 4
transl med aging: 4
j cachexia sarcopenia muscle: 4
j clin invest: 3
endocrinology: 3
free radic biol med: 3
am j physiol heart circ physiol: 3
dis model mech: 3
proc natl acad sci u s a: 3
sci adv: 3
jci insight: 3
nat metab: 3
mol brain: 2
aging pathobiol ther: 2
int j mol sci: 2
nature: 2
nutrients: 2
nat methods: 2
diabetes: 2
endocr relat cancer: 2
j neurochem: 2
j clin endocrinol metab: 2
cell: 2
mol neurodegener: 2
ebiomedicine: 2
faseb j: 2
am j primatol: 2
am j physiol endocrinol metab: 2
hum mol genet: 2
bmc bioinformatics: 2
exp gerontol: 2
plos genet: 2
front genet: 2
eneuro: 2
commun biol: 2
j alzheimers dis: 2
front aging: 1
transl psychiatry: 1
front rehabil sci: 1
nat cell biol: 1
radiology: 1
front med (lausanne): 1
front nutr: 1
mech ageing dev: 1
am j respir cell mol biol: 1
epigenetics chromatin: 1
front cell neurosci: 1
alzheimers dement: 1
j hepatol: 1
j exp biol: 1
j immunother cancer: 1
acs chem biol: 1
neuropharmacology: 1
mol neurobiol: 1
j clin psychiatry: 1
oncotarget: 1
j mol biol: 1
iscience: 1
neuron: 1
embo j: 1
am j respir crit care med: 1
j cereb blood flow metab: 1
cell stem cell: 1
j neurosci: 1
j periodontal res: 1
j proteome res: 1
aging dis: 1
j neuroinflammation: 1
angiology: 1

science: 1
exp hematol: 1
int j obes (lond): 1
j orthop: 1
function (oxf): 1
biochim biophys acta biomembr: 1
acta neuropathol: 1
cell syst: 1
oncoimmunology: 1
mol cell proteomics: 1
biochem biophys rep: 1
j vis exp: 1
brain behav: 1
mol genet metab: 1
methods cell biol: 1
ageing res rev: 1
bioinformatics: 1
arterioscler thromb vasc biol: 1
bmc genomics: 1
j expo sci environ epidemiol: 1
mol immunol: 1
j virol: 1
am j cardiol: 1
mol vis: 1
j am soc nephrol: 1
embo mol med: 1
bmc biol: 1
stem cell reports: 1
j am soc mass spectrom: 1
leukemia: 1
g3 (bethesda): 1
rna biol: 1
j cell biol: 1
neurobiol dis: 1
sci data: 1
circ res: 1
viruses: 1
front endocrinol (lausanne): 1
am j physiol cell physiol: 1
sci transl med: 1
diabetologia: 1
ann n y acad sci: 1
mol metab: 1
cancer cell: 1
npj aging mech dis: 1
lab invest: 1
chem sci: 1
hepatology: 1
virulence: 1
physiol genomics: 1
j cell sci: 1
nucleic acids res: 1
mol biol evol: 1
integr zool: 1
aging clin exp res: 1
addict biol: 1
plos biol: 1
front immunol: 1
comput methods programs biomed: 1
cancer res: 1
am j clin nutr: 1
stem cells: 1
genetics: 1
mol cell: 1
j mol endocrinol: 1
zoo biol: 1
am j physiol renal physiol: 1
clin biomech (bristol): 1
pathobiol aging age relat dis: 1
connect tissue res: 1
